# Impact of Mechanical and Manual Peeling on the Volatile Profile of White Pepper (*Piper nigrum* L.)

**DOI:** 10.3390/foods13152458

**Published:** 2024-08-03

**Authors:** Yuan Zhang, Peiyao Yu, Lijiao Wei, Bing Zhang, Dezhan Shen, Zhenhua Zhao, Xinbo Guo

**Affiliations:** 1 Agricultural Machinery Research Institute, Guangdong Engineering Technology Research Center of Precision Emission Control for Agricultural Particulates, Key Laboratory of Agricultural Equipment for Tropical Crops, Ministry of Agriculture and Rural Affairs, Chinese Academy of Tropical Agricultural Sciences, Zhanjiang 524091, China; agrizy@catas.cn (Y.Z.); shen_taoxmf@163.com (D.S.); 18634814019@163.com (Z.Z.); 2 School of Food Science and Engineering, Guangdong Province Key Laboratory for Green Processing of Natural Products and Product Safety, Engineering Research Center of Starch and Vegetable Protein Processing Ministry of Education, South China University of Technology, Guangzhou 510640, China; yupeiyao0118@126.com (P.Y.); dbkxydd@163.com (B.Z.)

**Keywords:** manual peeling, mechanical peeling, immersion, steaming, flavor

## Abstract

Mechanical peeling is more efficient and environmentally friendly compared to manual peeling. However, comparative studies on the quality of mechanically peeled pepper and manually peeled pepper are limited. This study utilized GC-MS to investigate the effects of immersion, steaming, and peeling machinery speed on the volatile composition of white pepper. A total of thirteen monoterpenes and seven sesquiterpenes were detected, with 3-carene, D-limonene, and sabinene being the most abundant monoterpenoids and β-caryophyllene, δ-elemene, and α-copaene being the most abundant sesquiterpenes. The total volatiles increased with longer steaming times and higher peeling machinery speeds. Compared to manual peeling or steaming followed by mechanical peeling, the volatile content of pepper was higher when using mechanical peeling alone. Additionally, relative odor activity values showed that 3-carene and D-limonene were the main contributors to flavor, with 3-carene, β-caryophyllene, and α-copaene being key volatiles responsible for flavor distinctions. This research aims to provide theoretical support for developing a superior and environmentally friendly mechanical method to replace manual labor.

## 1. Introduction

*Piperaceae* comprises five genera, including *Piper*, *Peperomia*, *Zippelia*, *Manekia*, and *Verhuellia* [1]. *Piper* is a well-known genus due to its unique flavor and therapeutic characteristics, which include antioxidant, anti-inflammatory, antibacterial, analgesic, and neuroprotective activities [2,3]. There are about 2000 species of *Piper*, one of which is *Piper nigrum* L. [4]. *Piper nigrum* L. is widely cultivated in tropical and subtropical regions such as India, Brazil, Malaysia, and China [5].

The fruit of *Piper* can be processed into white or black pepper depending on its maturity and processing method. Black pepper is made from dried immature fruit, while white pepper is made from ripe fruit that has been peeled off the flesh and then dried [6]. White pepper is valued more than black pepper due to its stronger pungency, milder flavor, lighter color, and higher bioactivity [7,8]. Various methods have been utilized in the production of white pepper, such as water retting [9], steaming [5], mechanical peeling [10], and solid-state fermentation [11]. Water retting has been widely used because of its high yield and low cost, especially in countries with large-scale white pepper production [7]. However, prolonged soaking of pepper in rivers can easily cause water pollution, produce foul odors, and result in higher microbial counts [10]. Additionally, water retting is often followed by manual foot stamping to complete the peeling process, which is time-consuming and labor-intensive. Mechanical peeling can not only greatly improve the efficiency of peeling but is also friendly to the environment, while there is a lack of comparative studies on the quality of white pepper produced by manual and mechanical peeling.

Due to the remarkable culinary and medicinal value of *Piper nigrum* L., the identification of its ingredients is of particular importance. Research has predominantly focused on its essential oil, comprising terpene hydrocarbons, oxygenated terpenes, and aromatic compounds [12,13,14]. Gas chromatography-mass spectrometry (GC-MS) has been widely utilized to analyze pepper’s volatile components. Ashokkumar et al. (2021) employed GC-MS to evaluate the chemical composition of essential oils from 18 black pepper accessions in southern India [13]. Similarly, Fan et al. (2020) employed GC-MS to analyze the volatile components of grafted and non-grafted white pepper [15].

Recent research has primarily focused on microbial community succession during the manual peeling process [11,16] and the invention of machinery for mechanical peeling [17,18]. However, there is a noticeable lack of studies comparing the quality differences between mechanically peeled pepper and manually peeled pepper. This study aims to address this gap by utilizing advanced analytical techniques, such as gas chromatography-mass spectrometry (HS-SPME-GC-MS), to systematically compare the volatile compositions of white peppers processed by various manual and mechanical peeling methods. This study reveals the effects of immersion, steaming, and mechanical processing on the volatiles and flavor profiles of white pepper and also provides theoretical support for finding a high-quality, efficient, and environmentally friendly pepper peeling method that can replace traditional manual peeling.

## 2. Materials and Methods

### 2.1. Chemicals

Sodium chloride (NaCl) was purchased from Sangong Biotech Co., Ltd. (Shanghai, China), S-(+)-2-octanol (98%) was purchased from Shanghai Yuanye Biotechnology Co., Ltd. (Shanghai, China), and n-alkane (C7-C30) of chromatographic grade was purchased from Sigma Aldrich (St. Louis, MO, USA).

### 2.2. Processes of Piper nigrum L.

The fresh pepper spikes of *Piper nigrum* L. were provided by the Agricultural Machinery Research Institute, Chinese Academy of Tropical Agricultural Sciences. All the pepper spikes used in the experiment were picked on the same day of peeling, and the picking standard was that the whole spikes contained more than 4 red fruits or more than 8 yellow fruits. Peppers were treated in three processing methods including mechanical peeling after steaming, mechanical peeling without steaming, and manual peeling after water immersion. For mechanical peeling after steaming, 120 g of peppers was averagely divided into three groups: 15 min (S-15), 20 min (S-20), and 25 min (S-25) of steaming at 100 °C, followed by peeling at 45 r/min. For mechanical peeling without steaming, 120 g of peppers was averagely divided into three groups based on peeling speed: 30 r/min (M-30), 45 r/min (M-45), and 60 r/min (M-60). For manual peeling after water immersion, 120 g of peppers was averagely divided into three groups based on immersion duration, 5 d (I-5), 7 d (I-7), and 10 d (I-10), followed by manual rubbing. The manual peeling after water immersion groups represented the control treatment. The 6TH-200 pepper peeling machine (Kunming Kanglixin Electronic Machinery Co., Ltd., Yunnan, China), with a power of 2.6 kW, was used for mechanical peeling, operating continuously for 2 min and 45 s. The steaming equipment 6SH-200 (Kunming Kanglixin Electronic Machinery Co., Ltd., Yunnan, China) was used in steaming, with a power of 2.56 kW, a steaming temperature of 100 °C, and a water consumption of 0.4 m^3^ per ton of pepper. As for the immersion process, the pepper-to-water ratio was 1:2.2, using tap water. The peeled peppers were dried by the LGJ-S30 drying machine (Beijing Sihuan Qihang Technology Co., Ltd., Beijing, China) at 65 °C for 7 h, to ensure their moisture content was reduced to below 14%.

### 2.3. Determination of Volatiles

The determination of volatiles was carried out by HS-SPME-GC-MS according to a previous report with modification [19]. A total of 2 mL of saturated sodium chloride solution containing S-(+)-2-octanol and 3 g of ground sample were added in a headspace bottle and equilibrated at 60 °C for 15 min. Volatiles were then adsorbed by a 50/30 µm of CAR/PDMS/DVB extraction fiber (Qingdao Zhenzheng Analytical Instrument Co., Ltd., Shandong, China) for 45 min and desorbed at 40 °C for 4 min. Volatiles were separated by DB-5MS column (30 m × 250 μm × 0.25 μm, Agilent Technologies, Palo Alto, CA, USA) and analyzed by a GC system (GC, Agilent Technologies 7890B, Palo Alto, CA, USA) equipped with a triple quadrupole-MS (TQ-MS, 7000C GC/MS Triple Quad, Agilent Technologies, Palo Alto, CA, USA). The temperature program was as follows: kept at 40 °C for 3 min, then increased to 100 °C at 5 °C·min^−1^, and finally increased to 280 °C at 15 °C·min^−1^ and held for 5 min. In terms of mass spectrometry conditions, the electronic energy of the EI mode was 70 eV with a mass scan range of 33–500 m/z, an ion source temperature of 230 °C, and a quadrupole temperature of 150 °C. S-(+)-2-octanol was selected as the internal standard. Each compound was identified using the NIST 20 standard mass spectrometry library (Agilent Technologies G1033A, Palo Alto, CA, USA). The concentrations of the volatile compounds were determined using Equation (1) provided by Rotsatchakul et al. (2008) [20].

(1) $$C_{i}\;=\;C_{is}\;\times \;\frac{A_{i}}{A_{is}}\;\times \;f_{i}$$

*C_i_* and *A_i_* are the concentration and peak area of compound *i*, respectively, and the concentration and peak area of the internal standard are *C_is_* and *A_is_*. As the correction factor of compound *i*, *f_i_* was calculated using the quantitative ion peak area of the internal standard and compound *i* standard. The data are reported as mean ± SD (*n* = 3) mg·g^−1^ DW.

### 2.4. Calculation of Relative Odor Activity Values (ROAVs)

To evaluate the contribution of individual compounds to the overall aroma, Equation (2) was used to calculate the relative odor activity values of all volatile compounds obtained from white pepper [21].

(2) $$ROVA_{i}\;=\;\frac{C_{i}}{T_{i}}\;\times \;\frac{T_{max}}{C_{max}}\;\times \;100$$

The *C_i_* represents the relative concentration of each volatile compound, the *T_i_* represents the odor threshold of each volatile compound measured in water (mg·g^−1^), and the *T_max_* and *C_max_* correspond to the maximum of *C_i_*/*T_i_* among all the volatile compounds in the same white pepper sample. The odor threshold of each volatile compound was obtained from the literature data reported by Cuevas-Glory [22], Multari [23], and Cui [24]. The ROAV ranges from 0 to 100. Volatile compounds with an *ROAV* ≥ 1.0 are key odor compounds, those with an *ROAV* between 0.1 and 1.0 modify aroma, and compounds with an *ROAV* < 0.1 are potential aroma contributors [25].

### 2.5. Statistical Analysis

Each sample was analyzed in triplicate and performed as mean ± SD mg·g^−1^ DW. Volatiles were identified using Agilent NIST 20. Partial least-squares discriminant analysis (PLS-DA) was plotted by MetaboAnalyst 5.0 (https://www.metaboanalyst.ca (accessed on 15 April 2024)). Pie chart, stacked bar chart, cluster heatmaps, and correlation plots were generated by Origin 2021 (Origin Lab Corporation, Northampton, MA, USA). One-way analysis of variance (ANOVA) followed by Duncan’s test was used to determine statistical significance with *p* < 0.05 by SPSS 26 (IBM Corp., Armonk, NY, USA) software.

## 3. Results

### 3.1. The Contents of Volatile Components

**Figure 1:**
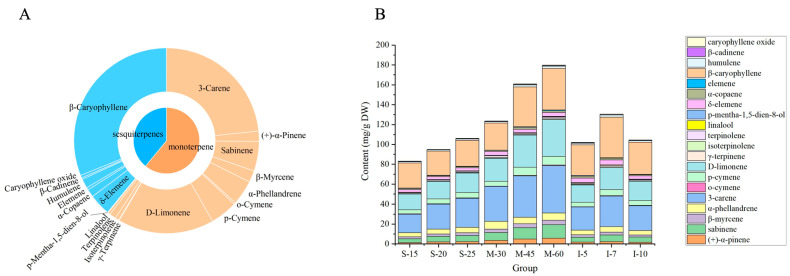
(**A**) Pie chart of volatile compounds in pepper. (**B**) Stacked bar chart of volatile compound contents in pepper under different treatments. S-15: mechanical peeling after steaming 15 min; S-20: mechanical peeling after steaming 20 min; S-25: mechanical peeling after steaming 25 min; M−30: 30 r/min mechanical peeling; M-45: 45 r/min mechanical peeling; M-60: 60 r/min mechanical peeling; I-5: manual peeling after 5 days of immersion; I-7: manual peeling after 7 days of immersion; and I-10: manual peeling after 10 days of immersion.

A total of twenty volatile compounds were detected by HS-SPME-GC-MS, including thirteen monoterpenes and seven sesquiterpenes (Figure 1A). The content of volatiles was shown in Table 1, and the stacked bar chart of volatile content was presented in Figure 1B. The β-caryophyllene, 3-carene, and D-limonene contents were the highest, consistent with Tran et al. (2020) [14]. Monoterpenes ranged from 60.39% to 72.11% across all groups, while sesquiterpenes ranged from 27.89% to 39.61%. M-60 had the highest monoterpene (128.17 ± 2.86 mg·g^−1^ DW) and sesquiterpene (51.63 ± 1.77 mg·g^−1^ DW) levels. In contrast, S-15 had the lowest monoterpenes (51.79 ± 3.39 mg·g^−1^ DW), and S-20 had the lowest sesquiterpenes (30.41 ± 2.41 mg·g^−1^ DW).

To investigate whether mechanical peeling can replace manual peeling, manual peeling with immersion in water (I-5, I-7, I-10) was regarded as the control groups. In the control groups, I-7 had the highest monoterpene and sesquiterpene contents (79.45 ± 7.72 mg·g^−1^ DW and 51.07 ± 4.56 mg·g^−1^ DW). The monoterpene and sesquiterpene contents of I-5 and I-10 were similar, with monoterpenes at 61.60 ± 7.04 mg·g^−1^ DW and 64.96 ± 0.64 mg·g^−1^ DW and sesquiterpenes at 40.41 ± 0.98 mg·g^−1^ DW and 39.47 ± 0.24 mg·g^−1^ DW, respectively. Mechanical peeling without steaming groups (M-30, M-45, and M-60) had higher total volatile component content than the mechanical peeling after steaming (S-15, S-20, and S-25) and control groups (I-5, I-7, and I-10). The total volatile component content of M-45 (160.87 ± 10.01 mg·g^−1^ DW) was significantly higher than that of mechanical peeling after steaming groups, which were all peeled at a speed of 45 r/min. In addition, the content of total volatiles increased with the extension of the steaming time and the increase in the peeling machine speed. However, there was no such pattern in the control groups, which were immersed in water for different days.

**Figure 2:**
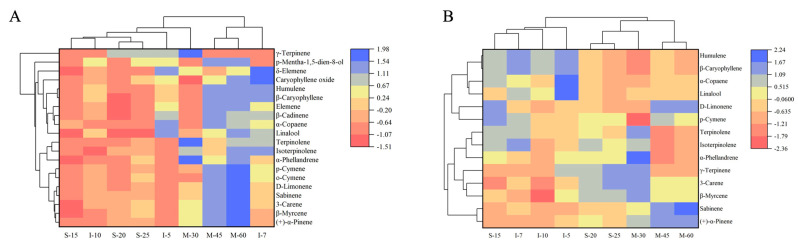
(**A**) The correlation cluster heat map of peppers and volatile compound contents. (**B**) The correlation cluster heat map of peppers and ROAVs of volatile compounds. S-15: mechanical peeling after steaming 15 min; S-20: mechanical peeling after steaming 20 min; S-25: mechanical peeling after steaming 25 min; M-30: 30 r/min mechanical peeling; M-45: 45 r/min mechanical peeling; M-60: 60 r/min mechanical peeling; I-5: manual peeling after 5 days of immersion; I-7: manual peeling after 7 days of immersion; and I-10: manual peeling after 10 days of immersion.

**Figure 3:**
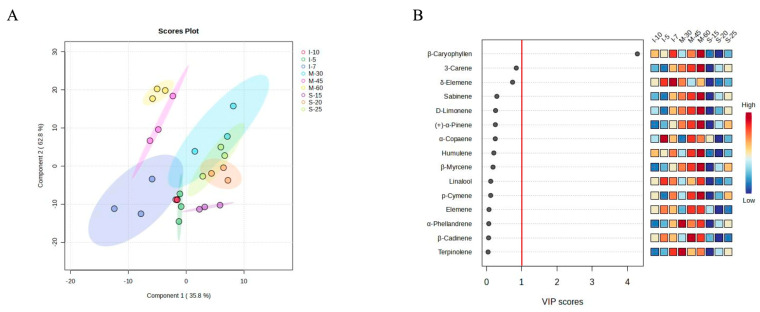
(**A**) The PLS-DA of peppers under different treatments based on volatile compound contents. (**B**) The VIP scores of volatile compounds derived from (**A**). This figure only demonstrates some of the volatile compounds with higher VIP scores. S-15: mechanical peeling after steaming 15 min; S-20: mechanical peeling after steaming 20 min; S-25: mechanical peeling after steaming 25 min; M-30: 30 r/min mechanical peeling; M-45: 45 r/min mechanical peeling; M-60: 60 r/min mechanical peeling; I-5: manual peeling after 5 days of immersion; I-7: manual peeling after 7 days of immersion; and I-10: manual peeling after 10 days of immersion.

To further comprehend the distinctions of volatile components in different samples, the cluster heatmap, which revealed the correlations between samples and volatiles contents, was drawn (Figure 2A). Mechanical peeling after steaming groups were similar to I-10, and mechanical peeling without steaming groups were similar to I-7, especially M-45 and M-60. The contents of α-pinene, sabinene, β-myrcene, 3-carene, o-cymene, p-cymene, and D-limonene increased with the peeling machine speed. To better distinguish the volatile composition of peppers under different processing conditions, PLS-DA was performed (Figure 3A). The 95% confidence intervals of M-30 and M-45 partially overlapped with I-7, and M-30 simultaneously overlapped with I-5, I-7, S-20, and S-25. M-30, M-45, and M-60 were clearly distinguishable. However, the mechanical peeling after steaming groups and manual peeling after water immersion groups could not be completely separated. The variable importance in projection (VIP) score of volatiles derived from PLS-DA is shown in Figure 3B. Compounds with VIP scores ≥ 1.0 were considered critical.

### 3.2. The ROAVs of Volatile Components

Among the total of twenty volatiles, eleven monoterpenes, and three sesquiterpenes had known odor thresholds. The contents of compounds without odor threshold were mostly below 0.5 mg·g^−1^ DW. To evaluate the aroma potency of each volatile compound in white pepper, the ROAVs of 14 characteristic volatiles with known odor thresholds were calculated (Table S1). The ROAV percentage of monoterpenes in all samples ranged from 89.96% to 94.65%, while that of sesquiterpenes ranged from 5.35% to 10.04%. S-15 showed the highest total ROAVs (181.48 ± 12.29), with monoterpenes also being the highest (165.07 ± 11.24). In contrast, M-30 had the lowest ROAVs of monoterpenes (155.04 ± 13.57) and sesquiterpenes (8.77 ± 0.96). Regardless of the treatment method, 3-carene exhibited the highest C_i_/T_i_ in all samples, making it the predominant contributor to the aroma. It constituted at least 55% of the flavor in each sample. D-limonene contributed about 21% to the flavor, while α-pinene, p-cymene, and β-caryophyllene each contributed approximately 5%. As the steaming time extended, the contribution of D-limonene to the sample’s flavor diminished.

**Figure 4:**
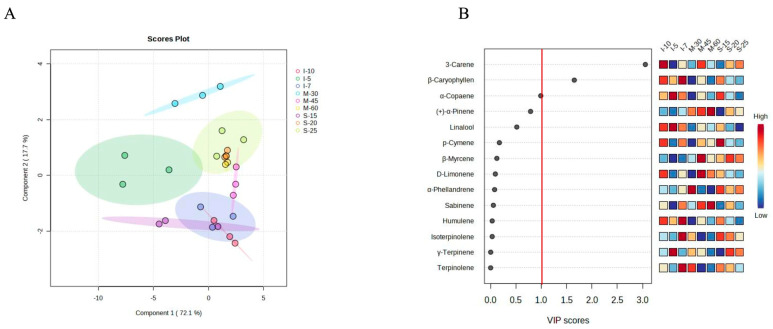
(**A**) The PLS-DA of peppers under different treatments based on ROAVs of volatile compounds. (**B**) The VIP scores of volatile compounds derived from (**A**). This figure only demonstrated some of the volatile compounds with higher VIP scores. S-15: mechanical peeling after steaming 15 min; S-20: mechanical peeling after steaming 20 min; S-25: mechanical peeling after steaming 25 min; M-30: 30 r/min mechanical peeling; M-45: 45 r/min mechanical peeling; M-60: 60 r/min mechanical peeling; I-5: manual peeling after 5 days of immersion; I-7: manual peeling after 7 days of immersion; and I-10: manual peeling after 10 days of immersion.

The cluster heatmap (Figure 2B) showed the correlations between samples and the ROAV percentages of each volatile. The flavor characteristics of S-15 were similar to the manual peeling groups, particularly I-7. In contrast, S-20 and S-25 were similar to the mechanical peeling without steaming groups. To elucidate the flavor distinctions among the samples, PLS-DA was performed (Figure 4A). The 95% confidence interval of S-15 partially overlapped with I-7 and I-10, while M-45 partially overlapped with I-7 and S-25. Additionally, S-20 and M-60 were entirely within the 95% confidence interval of S-25. The complete separation of M-30, M-45, and M-60 indicated significant flavor differences among these samples. The VIP scores of volatiles derived from PLS-DA (Figure 4A) highlighted that key volatiles contributing to the pepper flavor include 3-carene, β-caryophyllene, and α-copaene.

### 3.3. Correlation Analysis of Volatile Compounds

**Figure 5:**
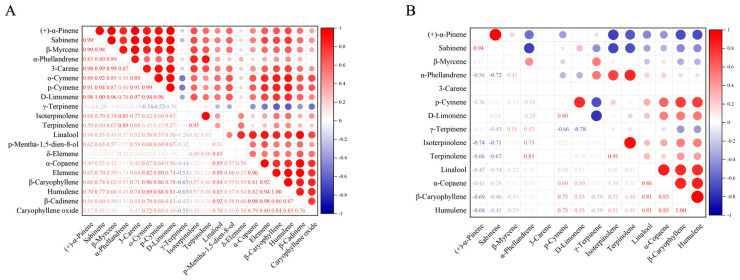
(**A**) The content−based correlation analysis of 20 volatile compounds. (**B**) The ROAV−based correlation analysis of 14 volatile compounds.

To better understand the relationship between volatiles, correlation analysis graphs were generated based on their contents and ROAVs, as depicted in Figure 5. Except for γ-terpinene, all compounds showed positive correlations with each other in the content-based correlation analysis (Figure 5A). Among monoterpenes, α-pinene, sabinene, β-myrcene, α-phellandrene, 3-carene, o-cymene, p-cymene, and D-limonene were highly correlated. Among sesquiterpenes, α-copaene, elemene, β-caryophyllene, humulene, β-cadinene, and caryophyllene oxide were strongly correlated. Notably, the correlation coefficient between D-limonene and sabinene reached 1.0, as did β-caryophyllene and humulene. While the ROAV-based correlation analysis (Figure 5B) demonstrated that most compounds showed negative correlations with α-pinene, sabinene, and γ-terpinene. Among monoterpenes, only α-pinene and sabinene, p-cymene and D-limonene, α-phellandrene and terpinolene, and isoterpinolene and terpinolene showed strong positive correlations. All sesquiterpenes displayed significant positive correlations. The correlation coefficient between β-caryophyllene and humulene remained consistent with Figure 5A, reaching 1.0.

## 4. Discussion

In terms of contents, the most abundant monoterpenoids were 3-carene, D-limonene, sabinene, p-cymene, and α-phellandrene. The most abundant sesquiterpenes included β-caryophyllene, δ-elemene, α-copaene, and humulene. According to the VIP scores from PLS-DA (Figure 3B), β-caryophyllene, 3-carene, and δ-elemene were the most critical volatiles for the volatile composition of pepper. In terms of ROAVs, the main monoterpenoids for pepper flavor were 3-carene, D-limonene, α-pinene, and p-cymene, while the main sesquiterpenes included β-caryophyllene and α-copaene. 3-carene showed an ROAV of 100 in all samples, meaning that it is the dominant flavor ingredient in white pepper, which provides a resinous aroma [22]. D-limonene had the second highest ROAV, giving a lemon-like flavor [22]. Resinous-flavored 3-carene [22], citrusy-flavored β-caryophyllene [22], and vegetable-flavored α-copaene [24] were identified as key volatiles responsible for flavor distinctions under different processing treatments. For mechanical peeling without steaming groups, the citrusy and vegetable flavors were the weakest, whereas for manual peeling after water immersion groups, the citrusy and vegetable flavors were the strongest. This suggested that the traditional manual method may be more effective in preserving or enhancing certain flavor profiles compared to mechanical methods.

### 4.1. Effect of Immersion on Volatile Compounds of Pepper

As pepper ripens, the levels of certain monoterpenoids, such as 3-carene and terpinolene, increase [26]. With the extension of immersion time, the ripeness of pepper increased and the terpenes accumulated, thus the terpenoid contents in I-7 were higher than I-5. Meanwhile, as the immersion time lengthens, pectin and cellulose in the pepper gradually hydrolyze [16], causing cell wall damage that may increase the volatilization or degradation of volatiles. Moreover, *Prevotella*, *Lactococcus*, *Selenomonas*, *Candida*, and *Fusarium* were identified as the predominant microbial communities during the immersion [16]. There may be some unexplored patterns between these microorganisms and volatile contents, which could account for the variations in volatile contents during the immersion. Pepper also contains several pungent-tasting alkaloids, including piperine, which is known for its antioxidant, anti-inflammatory, and bio-enhancing properties [27]. Prolonged immersion may cause substances like piperine to slowly dissolve into the water, potentially disrupting pepper’s metabolic processes and inhibiting terpenoid synthesis or causing their degradation. All the above may explain why terpenoid contents in I-10 were lower than in I-7.

Considering the overall flavor profile of the pepper, α-copaene was the only one among the three key volatiles that exhibits a notable difference across the three groups (I-5, I-7, I-10). α-copaene, characterized by a vegetable-like, hop-like, and woody scent [24], contains a carbon–carbon double bond, making it prone to oxidation. With prolonged immersion, α-copaene may undergo oxidation or isomerization, reducing its contribution to the overall flavor of pepper, so the ROAV of α-copaene was highest in I-5 and decreased drastically with longer immersion times. The flavor profile of manually peeled pepper after 5 days of immersion is slightly superior to that of pepper immersed for 7 and 10 days.

### 4.2. Effect of Mechanical Peeling on Volatile Compounds of Pepper

Both M groups (M-30, M-45, and M-60) and S groups (S-15, S-20, and S-25) underwent mechanical peeling, but S groups received steam treatment beforehand. D-limonene, 3-carene, sabinene, and p-cymene are all able to assist plants against environmental stress [28]. Pepper tissues subjected to mechanical peeling experienced more severe mechanical damage than those peeled manually. The faster the mechanical peeling speed, the greater the stress response, so M-60 the showed highest levels of D-limonene, 3-carene, sabinene, and p-cymene. This result was consistent with some existing studies on the effects of mechanical vibration on volatile compounds. Xu et al. (2021) [29] investigated the effect of mechanical vibration on the volatile compounds of blueberry fruit and speculated that vibration accelerated the ripening process by enhancing physiological metabolism, leading to an accumulation of alcohols. Similarly, Xu et al. (2020) [30] evaluated the effect of drop shock on the relative content of volatile compounds in apples and concluded that mechanical vibration stimulated the accumulation of aldehydes and esters.

The compositions of volatile compounds in M-45 and M-60 were similar to I-7, the ROAVs of volatile compounds in M-45 were similar to I-7. These observations suggested that mechanically peeled pepper can replace manually peeled pepper to some extent. However, it is crucial to consider pepper’s stress response to mechanical and thermal effects. For practical applications, a peeling speed of 45 r/min is recommended.

### 4.3. Effect of Steaming on Volatile Compounds of Pepper

In the mechanical peeling after steaming groups, all dominant monoterpenoids increased with the steaming time. Steaming is another form of environmental stress. Compounds like 3-carene, D-limonene, sabinene, and p-cymene can help plants cope with stress [28]. The stronger the environmental stimuli, the greater the production of compounds related to immune response. Moreover, the heat from steaming can break chemical bonds in the pericarp’s connective tissues, compromising its structural integrity [7], which may release loosely bound monoterpenes. Consequently, the monoterpene content increased progressively with longer steaming times. Gonzalo et al. (2022) [31] investigated the impact of thermal and mechanical treatments on the volatile aroma composition of seaweed (*Laminaria digitata*) and concluded that thermal processing prior to mechanical treatment softens the algae tissues, potentially enhancing the release of cellular compounds, including cell wall polysaccharides, phenolics, and lipids. They also noted that longer heating times can lead to the formation of aldehydes such as hexanal and heptanal, which are related to the degradation of fatty acids. Enzyme catalysis can convert certain aldehyde precursors into terpenoid compounds, which also helps explain why the terpenoid content increases with longer steaming times from another perspective.

The volatile compositions of S-15 and I-10 were remarkably similar, the overall flavor of S-15 was extremely comparable to I-7 and I-10. These observations illustrate the potential of mechanically peeled pepper after 15 min of steaming as an alternative to manually peeled pepper after 7 or 10 d of immersion. Prolonging the steaming time beyond 15 min resulted in a distinguishable flavor difference between mechanically and manually peeled pepper, indicating that prolonging the steaming time is not advisable. Further research is needed to determine if there is a more suitable and shorter time for pepper peeling. Additionally, thermal treatment may affect the color of white pepper and reduce the quantity of essential oil [7].

## 5. Conclusions

This study identified 3-carene, D-limonene, and β-caryophyllene as the most abundant volatiles in *Piper nigrum* L., with 3-carene and D-limonene being the primary contributors to its overall flavor. Additionally, 3-carene, β-caryophyllene, and α-copaene were key volatiles responsible for flavor distinctions under different processing methods. The total volatile contents increased as the steaming time and peeling machinery speed increased. Meanwhile, with the extension of immersion days, the volatile content increased initially and declined thereafter. The flavor profile of manually peeled pepper after 5 days of immersion is slightly superior to that of pepper immersed for 7 and 10 days. The flavor of mechanically peeled pepper at 45 r/min was similar to manually peeled pepper after 7 d of immersion. Furthermore, the flavor of mechanically peeled pepper at 45 r/min after 15 min of steaming was comparable to manually peeled pepper after 7 d or 10 d of immersion.

This research revealed the feasibility of mechanical peeling instead of manual peeling, providing theoretical support for developing a high-quality, efficient, and environmentally friendly method for pepper peeling. However, this study only revealed that mechanically peeled pepper could exhibit similar flavors to manually peeled ones, but it did not indicate that the flavor quality of mechanically peeled pepper was superior to manually peeled ones and lacked sensory evaluation results to support these findings.

## Figures and Tables

**Table 1:** The contents of volatile compounds identified in *Piper nigrum* L. under different treatments (mg/g DW).

NO.	Compound	Type	RT (min)	S-15	S-20	S-25	M-30	M-45	M-60	I-5	I-7	I-10
C1	(+)-α-pinene	monoterpene	9.804	1.32 ± 0.04 f	2.18 ± 0.17 de	2.36 ± 0.14 d	3.52 ± 0.60 c	4.97 ± 0.35 b	5.83 ± 0.12 a	1.91 ± 0.27 de	2.34 ± 0.13 d	1.73 ± 0.02 ef
C2	sabinene	monoterpene	11.279	3.85 ± 0.20 g	5.40 ± 0.43 ef	5.95 ± 0.29 de	7.93 ± 0.95 c	11.42 ± 0.80 b	13.45 ± 0.15 a	4.93 ± 0.84 f	6.75 ± 0.25 d	5.19 ± 0.03 ef
C3	β-myrcene	monoterpene	11.699	1.91 ± 0.14 g	2.47 ± 0.21 def	2.83 ± 0.25 d	3.46 ± 0.31 c	4.01 ± 0.27 b	4.49 ± 0.10 a	2.31 ± 0.28 efg	2.67 ± 0.28 de	2.15 ± 0.01 fg
C4	α-phellandrene	monoterpene	12.244	4.06 ± 0.32 d	4.81 ± 0.36 d	5.55 ± 0.29 c	7.83 ± 0.59 a	6.39 ± 0.55 b	7.43 ± 0.12 a	4.74 ± 0.53 d	5.65 ± 0.54 c	4.47 ± 0.05 d
C5	3-carene	monoterpene	12.319	18.79 ± 1.43 g	25.29 ± 1.98 ef	29.17 ± 2.27 de	34.96 ± 3.05 c	41.52 ± 3.12 b	47.47 ± 0.72 a	23.30 ± 3.03 f	30.56 ± 2.83 d	24.91 ± 0.14 f
C6	o-cymene	monoterpene	12.683	0.29 ± 0.01 e	0.29 ± 0.02 e	0.35 ± 0.01 d	0.30 ± 0.02 e	0.50 ± 0.05 b	0.57 ± 0.03 a	0.30 ± 0.03 e	0.41 ± 0.04 c	0.31 ± 0.02 de
C7	p-cymene	monoterpene	12.848	4.24 ± 0.21 d	4.62 ± 0.35 cd	5.25 ± 0.24 c	4.79 ± 0.33 cd	8.28 ± 0.58 a	8.82 ± 0.73 a	4.44 ± 0.39 cd	6.19 ± 0.55 b	4.99 ± 0.22 cd
C8	D-limonene	monoterpene	13.002	15.62 ± 0.97 f	17.46 ± 1.34 ef	19.64 ± 1.05 de	23.14 ± 1.67 c	32.18 ± 2.46 b	36.95 ± 0.63 a	17.27 ± 1.55 ef	22.01 ± 2.63 cd	19.13 ± 0.10 e
C9	γ-terpinene	monoterpene	13.938	ND	0.16 ± 0.01 c	0.16 ± 0.00 bc	0.24 ± 0.02 a	ND	ND	0.17 ± 0.00 b	ND	ND
C10	isoterpinolene	monoterpene	14.675	0.54 ± 0.02 c	0.62 ± 0.04 bc	0.64 ± 0.02 bc	0.83 ± 0.05 a	0.69 ± 0.01 b	0.87 ± 0.09 a	0.54 ± 0.02 c	0.85 ± 0.16 a	0.52 ± 0.02 c
C11	terpinolene	monoterpene	14.799	0.90 ± 0.03 c	0.99 ± 0.08 bc	1.10 ± 0.04 bc	1.64 ± 0.09 a	1.18 ± 0.01 b	1.45 ± 0.10 a	0.97 ± 0.04 c	1.48 ± 0.29 a	0.95 ± 0.03 c
C12	linalool	monoterpene	15.262	0.27 ± 0.02 d	0.29 ± 0.02 d	0.30 ± 0.02 d	0.40 ± 0.02 c	0.49 ± 0.03 b	0.56 ± 0.03 a	0.56 ± 0.05 a	0.54 ± 0.02 a	0.44 ± 0.03 bc
C13	p-mentha-1,5-dien-8-ol	monoterpene	16.772	ND	ND	0.16 ± 0.01 c	ND	0.24 ± 0.00 b	0.28 ± 0.04 a	0.16 ± 0.01 c	ND	0.17 ± 0.00 c
C14	δ-elemene	sesquiterpene	19.377	2.71 ± 0.30 d	2.93 ± 0.27 cd	3.03 ± 0.16 cd	3.93 ± 0.23 b	3.36 ± 0.23 c	3.86 ± 0.28 b	4.48 ± 0.10 a	4.88 ± 0.49 a	3.40 ± 0.02 c
C15	α-copaene	sesquiterpene	19.866	1.30 ± 0.03 c	1.06 ± 0.04 d	1.12 ± 0.04 cd	1.08 ± 0.08 d	2.03 ± 0.16 a	1.98 ± 0.17 ab	2.05 ± 0.06 a	1.81 ± 0.21 b	1.24 ± 0.01 cd
C16	elemene	sesquiterpene	19.994	0.37 ± 0.01 d	0.33 ± 0.03 d	0.35 ± 0.02 d	0.36 ± 0.05 d	0.63 ± 0.04 a	0.64 ± 0.03 a	0.55 ± 0.01 b	0.55 ± 0.05 b	0.43 ± 0.00 c
C17	β-caryophyllene	sesquiterpene	20.368	25.05 ± 2.16 c	24.30 ± 1.94 c	26.15 ± 1.76 c	27.17 ± 3.36 c	39.95 ± 1.21 a	41.96 ± 1.17 a	30.88 ± 0.72 b	40.74 ± 3.53 a	32.16 ± 0.18 b
C18	humulene	sesquiterpene	20.724	1.33 ± 0.13 c	1.27 ± 0.11 c	1.36 ± 0.11 c	1.42 ± 0.19 bc	2.17 ± 0.09 a	2.26 ± 0.08 a	1.62 ± 0.04 b	2.13 ± 0.22 a	1.64 ± 0.02 b
C19	β-cadinene	sesquiterpene	21.267	0.23 ± 0.01 bc	0.19 ± 0.00 d	0.21 ± 0.02 cd	0.24 ± 0.05 bc	0.40 ± 0.01 a	0.39 ± 0.02 a	0.39 ± 0.02 a	0.36 ± 0.03 a	0.26 ± 0.00 b
C20	caryophyllene oxide	sesquiterpene	21.904	0.32 ± 0.03 e	0.33 ± 0.02 e	0.39 ± 0.04 d	0.23 ± 0.01 f	0.46 ± 0.04 c	0.54 ± 0.02 b	0.44 ± 0.03 c	0.60 ± 0.03 a	0.34 ± 0.01 e
total monoterpenes				51.68 ± 3.39	64.43 ± 5.01	73.35 ± 4.63	88.94 ± 7.70	111.75 ± 8.23	128.00 ± 2.86	61.53 ± 7.04	79.38 ± 7.72	64.92 ± 0.67
total sesquiterpenes				31.26 ± 2.67	30.41 ± 2.41	32.56 ± 2.15	34.36 ± 3.97	48.95 ± 1.78	51.57 ± 0.77	40.33 ± 0.98	51.03 ± 4.56	39.41 ± 0.24
total volatiles				82.94 ± 6.06	94.84 ± 7.42	105.91 ± 6.78	123.30 ± 11.67	160.70 ± 10.01	179.57 ± 3.63	101.86 ± 8.02	130.41 ± 12.28	104.33 ± 0.91

ND, not detected. Different letters represent significant (*p* < 0.05) differences according to ANOVA combined with Duncan’s multiple range test.

## Data Availability

The original contributions presented in the study are included in the article/supplementary material, further inquiries can be directed to the corresponding authors.

## Supplementary Materials

The following supporting information can be downloaded at: https://www.mdpi.com/article/10.3390/foods13152458/s1; Table S1: The ROAVs of volatile components in *Piper nigrum* L. under different treatments.
